# Supplementary material for: The nematode Caenorhabditis elegans enhances tolerance to landfill leachate stress by increasing trehalose synthesis
Source: PeerJ. 2024 May 22;12:e17332. doi: 10.7717/peerj.17332 (PMC11127639; doi:10.7717/peerj.17332)
Supplement: Supplemental Information 1 [file peerj-12-17332-s001.zip › Raw data with description/fig1/fig 1 data description.docx]

Data Description

Content: Figure 1 - Survival rate of nematodes exposed to landfill leachate treated with different processes

Factors: Time, Exposure conditions

Quantity: Data divided into four groups - CK, RAW, MBR, NFRO, with four replicates each day

Usage: For subsequent analysis, use data from the first three days, i.e., 72 hours of exposure.
