# Supplementary material for: The nematode Caenorhabditis elegans enhances tolerance to landfill leachate stress by increasing trehalose synthesis
Source: PeerJ. 2024 May 22;12:e17332. doi: 10.7717/peerj.17332 (PMC11127639; doi:10.7717/peerj.17332)
Supplement: Supplemental Information 1 [file peerj-12-17332-s001.zip › Raw data with description/fig4/fig 4 data description.docx]

Data Description

Content: Figure 4 - Carbohydrates of *Caenorhabditis elegans* treated with different landfill leachates.

Factors: Exposure conditions

Quantity: Data divided into four groups - CK, RAW, MBR, NFRO, with four replicates every group, every sample have three replicates.

Usage: Use the standard curve to count the concentration of different carbohydrates and protein. Apart from that, we need to use the obtained concentration values and the corresponding molar mass of sugars to calculate the molar concentration, and finally use protein concentration for correction to calculate the concentration of different sugars contained in each unit of protein concentration.
