# Supplementary material for: The nematode Caenorhabditis elegans enhances tolerance to landfill leachate stress by increasing trehalose synthesis
Source: PeerJ. 2024 May 22;12:e17332. doi: 10.7717/peerj.17332 (PMC11127639; doi:10.7717/peerj.17332)
Supplement: Supplemental Information 1 [file peerj-12-17332-s001.zip › Raw data with description/fig5/fig 5 data description.docx]

Data Description

Content: Figure 5 - Expression of genes related to trehalose metabolic pathway in *Caenorhabditis elegans* treated with different landfill leachates.

Factors: Exposure conditions

Quantity: Data divided into four groups - CK, RAW, MBR, NFRO, with three replicates every group, each sample is tested for internal reference gene *ACT-1* and detection genes

Usage: The CT data obtained from qRT-PCR was analyzed using the 2^-△△CT^ method. The specific calculation formula for 2^-△△CT^ is as follows:

2^-△△CT^=2^- [(CT experimental group CT- experimental act-1) - (CT control group - CT control act-1)]^

^Control refers to CK group; experimental refers to experimental groups including MBR, NFRO and RAW group.^
